# Secondary Metabolites with Biomedical Applications from Plants of the Sarraceniaceae Family

**DOI:** 10.3390/ijms23179877

**Published:** 2022-08-30

**Authors:** Ileana Miclea

**Affiliations:** Department of Fundamental Sciences, Faculty of Animal Science and Biotechnology, University of Agricultural Sciences and Veterinary Medicine, 400372 Cluj-Napoca, Romania; ileana.miclea@usamvcluj.ro

**Keywords:** Sarraceniaceae, secondary metabolites, biological activity

## Abstract

Carnivorous plants have fascinated researchers and hobbyists for centuries because of their mode of nutrition which is unlike that of other plants. They are able to produce bioactive compounds used to attract, capture and digest prey but also as a defense mechanism against microorganisms and free radicals. The main purpose of this review is to provide an overview of the secondary metabolites with significant biological activity found in the Sarraceniaceae family. The review also underlines the necessity of future studies for the biochemical characterization of the less investigated species. *Darlingtonia*, *Heliamphora* and *Sarracenia* plants are rich in compounds with potential pharmaceutical and medical uses. These belong to several classes such as flavonoids, with flavonol glycosides being the most abundant, monoterpenes, triterpenes, sesquiterpenes, fatty acids, alkaloids and others. Some of them are well characterized in terms of chemical properties and biological activity and have widespread commercial applications. The review also discusses biological activity of whole extracts and commercially available products derived from Sarraceniaceae plants. In conclusion, this review underscores that Sarraceniaceae species contain numerous substances with the potential to advance health. Future perspectives should focus on the discovery of new molecules and increasing the production of known compounds using biotechnological methods.

## 1. Introduction

Sarraceniaceae, part of the Ericales order, is a family of carnivorous plants native to the American continent. Biogeographic analyses reveal that the family originated in South America 44–53 Mya, and that by 25–44 Mya it had achieved a widespread distribution across South and North America [1]. Currently, the three genera that make up this family are *Darlingtonia*, *Heliamphora* and *Sarracenia* [2]. Darlingtonia is a monotypic genus endemic to the serpentine seeps and interdunal wetlands found from the northern coast of Oregon to California’s central Sierra Nevada. The highest density of documented sites is found in northwestern California in the Klamath, Siskiyou, Shasta and Trinity Mountains [1,3]. The tropical genus *Heliamphora* comprises ca. 23 species which grow in the Guaiana Highlands of northern Brazil, western Guyana and southern Venezuela [2,4]. They are only found in the Pantepui region where the tepuis, or sandstone table-top mountains, rise above the surrounding lowlands and they are generally confined to the tepui summits at elevations above 1500 m [5,6]. *Sarracenia* is the most widespread genus of the family, with ca. 11 species and a multitude of subspecies, varieties and hybrids which range from the Gulf Coast of Texas, Louisiana, Mississippi, Alabama and Florida, north along the Atlantic Coast to Newfoundland and Labrador and west through the northern Midwestern United States and southern Canada to eastern British Columbia. They populate bogs, savannas and wetland habitats where the soil has high organic content and high acidity but almost no nutrient availability [1,7].

Sarraceniaceae plants have adapted to life in nutrient-poor habitats because of the ability to supplement their diet with nutrients of animal origin. In order to lure their prey, leaves are converted into pitcher-shaped structures that attract, trap, digest and absorb nutrients from a variety of animal prey, mainly arthropods but also small vertebrates [2,8]. The arsenal for insect entrapment includes physical weapons such as downward pointing hairs, waxy surfaces (*Heliamphora*, *Sarracenia*) or leaf architecture that confuses the prey (*Darlingtonia*, *S*. *psittacina*) [9] in conjunction with chemical weapons represented by secondary metabolites. Plant secondary metabolites are a very diverse group of substances with over 500,000 identified members produced by secondary metabolic pathways. They make plants competitive in their own environment by exerting a wide range of effects on the plant itself and on other organisms. They play a variety of functions in plant growth and development (maintain perennial growth, induce flowering, fruit set and abscission), act as antimicrobials, repel pests or pathogens or attract numerous organisms from symbiotic microbes to pollinators [10,11]. In the context of carnivorous plant ecology, secondary metabolites play an important role in plant evolutionary adaptation [12] and are involved the four key stages of the carnivorous habit: prey attraction, capture, digestion and assimilation of nutrients [13]. Extrafloral nectar lures insects and also establishes slippery surfaces on the pitcher lip (*Heliamphora*) [14,15]. Other metabolites such as volatiles attract insects [16,17,18] while pigments create striking patterns both in the visible and UV light spectrums [19,20]. Compounds with chemopreventive and therapeutic potential belonging to three main groups (naphthoquinones, flavonoids, phenolic acids) have already been isolated from carnivorous plants of several genera, mainly *Dionaea*, *Drosera* and *Nepenthes* [21,22]. These substances are recognized as having huge potential for prevention and treatment of various diseases such as diabetes, cancer, neurodegenerative disease, cardiovascular disease and atherosclerosis [23].

Sarraceniaceae plants have been of horticultural interest for well over one hundred years which has often put them at risk of being poached [24]. Their significance also stems from being used for centuries to prepare medicine, particularly by people indigenous to the area they occupy. In Canada, *Sarracenia purpurea* (purple pitcher plant) has a long history of use as a traditional medicine against diabetes [25], gynecological issues, various conditions of the digestive tract, such as constipation or dyspepsia, and liver and kidney complaints [26]. It was employed as treatment for smallpox and other infectious diseases during the 19th century [27]. More recently, its activity and properties against herpes simplex virus type-1 (HSV-1) were explored and characterized. These studies show that *S. purpurea* extracts have antiviral activity against pox and herpes viruses by inhibiting free virion or viral attachment to host cells, as well as inhibiting the expression of viral gene transcription [27,28]. The healing properties of extracts observed by traditional medicine and the biological activity reported by recent research are probably due to the presence of secondary metabolites.

An analgesic effect has been proposed for *S. purpurea* aqueous extracts commercially available under the names Sarapin and P-Bloc. Use of the former in injections for regional analgesia in neuropathic pain [29] is supported by proof that it can be an effective modality of managing chronic, persistent low back pain [30]. In contrast, P-Bloc does not display a local anesthetic effect in a horse model, but could have a nonlocal anesthetic mechanism of action [31]. The same result was reported for Sarapin in a similar model [32] and in a double-blind trial with 500 consecutive human patients [33].

*Sarracenia* is the most studied genus of the family [31,34,35,36,37] but research has also been conducted on the metabolic profile of *Darlingtonia* [9] and the role of metabolites and extracts from three *Heliamphora* species. The iridoid sarracenin from *H. heterodoxa* and *H. tatei* is the major volatile compound used as insect attractant [17]. *H. nutans* extract has antiproliferative activity against lung tumor cells [38].

Given the current interest in carnivorous plants and substances with chemopreventive and therapeutic potential, the present review aims to provide an overview of the secondary metabolites with significant biological activities which can be isolated from *Darlingtonia*, *Heliamphora* and *Sarracenia* plants and employed to promote human and animal health. Sarraceniceae plants are rich in useful compounds and a second objective is to employ this synthesis of published literature as an opportunity for exploring the potential pharmaceutical and medical uses of the currently known metabolites. Its purpose is also to highlight the need for further research into the chemical composition of the barely investigated *Heliamphora* genus and the incompletely researched *Darlingtonia* and *Sarracenia* genera. Studies focusing on the extraction and identification of secondary metabolites from Sarraceniaceae plants were identified through a systematic search of Google Scholar and the MEDLINE database via the PubMed interface using search terms such as “metabolite” and the names of the three genera. A table was generated (Table 1) to ascribe each compound to a chemical class which helped name the main sections of the text. Afterwards, information about the identified metabolites was gathered by searching for the name or alternate names of each one (e.g., quercetin-3-O-rutinoside or rutin) accompanied by the term “biological activity” and its synonyms. Reference lists of relevant journal articles were searched in case papers had not been previously identified.

## 2. Flavonoids

**Figure 1:**
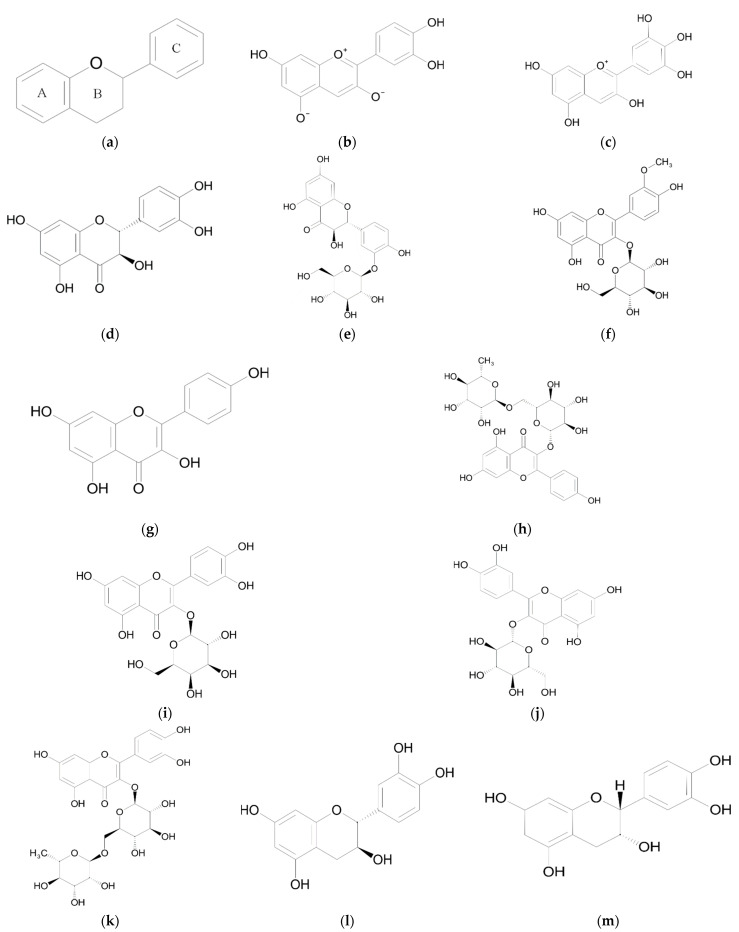
Chemical structures of flavonoids and flavonoid glycosides from Sarraceniaceae species: (**a**) basic flavonoid structure; (**b**) cyanidin; (**c**) delphinidin; (**d**) taxifolin; (**e**) taxifolin-3-O-glucoside; (**f**) isorhamnetin-3-O-glucoside; (**g**) kaempferol; (**h**) kaempferol-3-O-rutinoside; (**i**) quercetin-3-O-galactoside; (**j**) quercetin-3-O-glucoside; (**k**) quercetin-3-O-rutinoside; (**l**) (+)-catechin; (**m**) (−)-epicatechin.

Flavonoids belong to a class of plant-derived secondary metabolites with low molecular weight and a variable polyphenolic structure. They have the C6–C3–C6 general structural backbone in which the two C6 units (Ring A and Ring B) are of phenolic nature (Figure 1). Due to the hydroxylation pattern and variations in the chromane ring (Ring C), flavonoids can be further divided into different sub-groups such as anthocyanins, chalcones, flavanones, flavones, flavonols, isoflavonoids and flavan-3-ols (flavanols). The basic structures of flavonoids are aglycones but in plants most of them exist as glycosides. Their biological activities are dependent on both the structural difference and the glycosylation patterns [49,50]. Flavonoids are responsible for the color of fruits and flowers. Throughout the evolution of terrestrial plants, flavonoids have helped them gain frost hardiness and drought resistance and acted as UV filters and antimicrobial defense compounds [51,52]. Flavonoids are naturally found in fruits, vegetables, grains and certain beverages such as tea, cocoa and wine [53]. They are added to foods and food supplements but have also become an indispensable component of nutraceutical, pharmaceutical, medicinal and cosmetic applications [50]. The great interest in flavonoids stems from their anti-inflammatory, antimicrobial, antioxidant and antitumor properties [54,55]. Flavonoids are distributed in all *Sarracenia* leaf parts with the highest content being found in the operculum, the topmost part of the leaf, where they create variegation which lures insects and protect plant cells from light-produced free radicals [37,56].

### 2.1. Anthocyanidins

Anthocyanins are a subgroup of flavonoids that have the C6-C3-C6 flavan or 2-phenylbenzodihydorpyrane skeleton (Figure 1) [57]. More than 700 anthocyanins have been identified in nature [58]. In plants, they provide various colors such as red, pink, blue and purple and play key roles in plant reproduction by attracting pollinators and seed dispersers. Anthocyanins also help protect plants against abiotic and biotic stresses such as pathogens and predators, ultraviolet radiation, reactive oxygen species (ROS) and climate conditions [59,60]. Anthocyanins can be divided based on their substitutions, especially at Ring B, or the glycosylation at Rings A and C of the skeleton. The main sugars are the monosaccharides glucose, arabinose, galactose or the disaccharide rutinose (6-O-α-L-rhamnosyl-D-glucose) [57,61]. The color and stability, both chemical and physiological, of these pigments are influenced by pH, light, temperature and structure such as glycosylation and acylation patterns [62,63]. Anthocyanins without a sugar attached are called anthocyanidins. Cyanidin and delphinidin are the two most common anthocyanidins found in plant tissues with proportions of 50% and 12%, respectively [64].

High concentrations of anthocyanins are present in the operculum and upper leaf part of several *Sarracenia* hybrids [37]. Cyanidin is the main anthocyanidin detected in leaves of *S. flava, S. leucophylla, S. psittacina, S. purpurea* and *S. rubra* and in flowers of *S. rubra* and *S. leucophylla* [34]. Blue pigments detected in *Sarracenia* flowers were later identified as delphinidin glycosides [65].

A large quantity of delphinidin is present in *S. purpurea* petals (44%) and a smaller one (11%) in *S. psittacina flowers* [34]. Identification of minor anthocyanidins and of the sugars attached to the major ones requires further study.

#### Biological Activity

Anthocyanins in general and cyanidin and delphinidin in particular exhibit a broad range of pharmacological activities such as antioxidant [66], anticancer [67], antiobesity [68], cardioprotective, neuroprotective [57,69] and antidiabetic [63]. That is why several patents have been granted for research related to delphinidin use in cosmetics, anticancer therapy or as an antimicrobial agent [70]. Cyanidin and its glycosides display neuroprotective effects both in cells cultured in vitro [71] and in rats [72]. Cyanidin is part of a recently defined class of cancer chemopreventive agents called antimetastatic agents [73]. Delphinidin shows anticancer activity against cells from a variety of cancers such as breast, ovarian, colon, prostate, lung, hepatic, bone, blood and skin. In most cancers, it interferes with protein targets of the phosphatidylinositol 3 kinase/protein kinase B/mechanistic target of rapamycin (PI3K/Akt/mTOR) and mitogen-activated protein kinase (MAPK) signaling pathways [70]. It has also been shown to act against ovarian cancer synergistically with agents such as cisplatin and paclitaxel [74]. Both cyanidin and delphinidin have chemopreventive action against skin cancer [75]. Delphinidin displays cardioprotective and antihypertensive activity by reducing cardiac hypertrophy, cardiac dysfunction and oxidative stress [76]. High doses of anthocyanins have the potential to modulate carbohydrate metabolism and blood glycemic levels, and help reduce cardiovascular risk factors [77]. Delphinidin is reported to be a potent inhibitor of osteoclast differentiation and considered an effective agent for preventing bone loss in women with postmenopausal osteoporosis [78]. It can also reduce muscle atrophy in mice [79] while cyanidin has potential for treating patients with rheumatoid arthritis [80]. Delphinidin acts directly on viral particles of the hepatitis C virus and impairs their attachment to the cell surface [81]. It was also found to be effective against certain strains of the Zika virus [82] and to cure *Staphylococcus aureus* infection [83].

### 2.2. Flavonols, Flavonol Glycosides and Flavononol Glycosides

Flavonols are a class of flavonoids that have the 3-hydroxyflavone backbone with a double bond at the 2–3 position and a hydroxyl group at the 3 position. Despite being low molecular weight compounds, they present a great variability in terms of structural features [84]. In higher plants, flavonols are widely distributed in glycosylated form, with the most abundant being O-glycosides [85,86]. 

The major flavonoid components (Figure 1) are similar in all *Sarracenia* species with some variation in the “rubra complex” [87]. Although metabolite profiling has recently been carried out for a large number of accessions belonging to several species [9], the most studied members of the genus with respect to chemical composition are *S. flava* [16,18,46,47] and *S. purpurea*. Based on in vitro assays *S. purpurea*, is considered among the most biologically active species traditionally used by the indigenous Cree of James Bay (Canada) against diabetes [88]. Water and ethanol extracts from *S. purpurea* leaves and roots contain flavonols and flavonol glycosides together with flavan-3-ols (Table 1) [26,39,40]. The most abundant flavonol glycoside is taxifolin-3-O-glucoside with 90.659 mg/g in the ethanol extract and 39.094 mg/g in the water extract, followed by quercetin-3-O-galactoside with 34.833 mg/L and 20.784 mg/L, respectively [40]. Quercetin-3-O-galactoside is known to be one of the active principles of *Vaccinium vitis-idaea* berries [89].

#### 2.2.1. Biological Activity of *S. purpurea* Extracts

*S. purpurea* ethanol extract is more potent than metformin in increasing glucose uptake in C2C12 mouse muscle cells under basal and insulin-stimulated conditions. Isorhamnetin-3-O-glucoside, kaempferol-3-O-(6″-caffeoylglucoside) and quercetin-3-O-galactoside are believed to be the active principles which potentiate glucose uptake in vitro [26].

*S. purpurea* ethanol extract activates the AMP-activated protein kinase (AMPK) pathway and augments the expression of glucose transporter type 4 (GLUT4). The water extract intensifies glucose uptake by activating the insulin pathway that involves Akt. Quercetin-3-O-galactoside and quercetin 3-O-α-L-arabinopyranoside are biomarkers for the water extract that stimulates glucose uptake [35,90]. The ethanol plant extract also reduces activity of glucose-6-phosphatase, which is a key enzyme in gluconeogenesis and increases glucose storage by stimulating glycogen synthase activity. These actions result in a reduction in glucose production by rat hepatoma cells [26]. The extract also protects PC12 rat neuronal cells from death caused by hyper- or hypoglycemic conditions with the active compounds being quercetin-3-O-galactoside and the irridoid morroniside [41]. Therefore, it can be said that *S. purpurea* extract lowers blood glycaemia and protects against complications of diabetes [26]. However, it can potentially be harmful to renal cells such as the MDCK cell line by enhancing the activity of caspases and inducing apoptosis [91].

**Figure 2:**
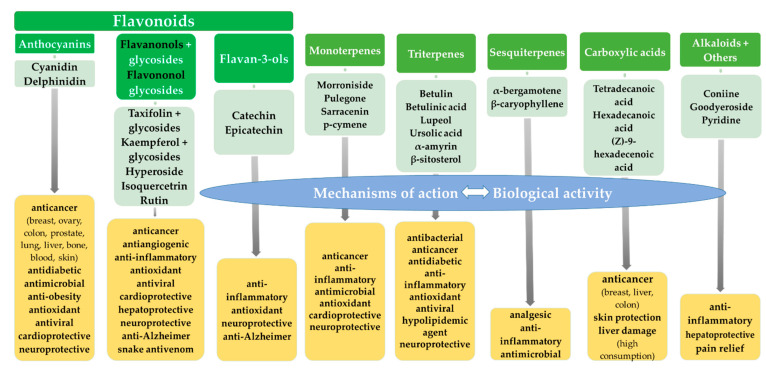
Biological activity of secondary metabolites from Sarraceniaceae plants.

The biological activity of *S. purpurea* extracts is partly due to the presence of various types of flavonoids (Figure 2). Some are the focus of intense research and their multiple biomedical effects have resulted in the production of drugs and dietary supplements while others are less investigated.

#### 2.2.2. Biological Activity of Taxifolin

Taxifolin (dihydroquercetin) or 5,7,3′,4′-flavan-on-ol is a flavanonol with two stereocenters on the C-ring and methylation of C-3, C-5 and C-7 [92]. Its glycosides can be found in medicinal plants such as *Hypericum* *perforatum* [93]. In in vitro and in vivo studies, taxifolin displays a wide range of health-promoting effects and biological activities such as antioxidant, anti-inflammatory, anti-Alzheimer, anticancer and antiangiogenic.

The structural orientation of taxifolin makes this compound capable of scavenging free radicals which corresponds to its antioxidant efficacy [94]. As shown by in vitro bioassays, when compared with standard antioxidant compounds such as butylated hydroxyanisole (BHA), butylated hydroxytoluene (BHT) α-tocopherol or Trolox, taxifolin has marked antioxidant, reducing ability, radical-scavenging and metal-chelating activities. This is why taxifolin can be used for minimizing or preventing lipid oxidation in food or pharmaceutical products which results in maintaining product nutritional quality and prolonging shelf life [95]. Taxifolin antioxidant activity is associated with blood capillary protection [96] and it is also exhibited through its neuroprotective effects via the inhibition of oxidative neuronal injuries in rat cortical cells [97].

Taxifolin can regulate the activation of nuclear factor kappa B (NF-κB) in rats diagnosed with cerebral ischemia–reperfusion injury. Furthermore, it is also associated with the suppression of leukocyte infiltration and inhibits the expression of cyclooxygenase-2 (COX-2) and inducible nitric oxide synthase (iNOS) in the brain [98]. In vitro, it represses osteoclastogenesis through the receptor activator of nuclear factor kappa Β ligand (RANKL). RANKL-induced gene expression is suppressed by taxifolin without significant cytotoxicity, a finding which is supported by bone loss prevention in an in vivo mouse model [99]. Taxifolin inhibits degranulation, generation of leukotriene C4 (LTC4), production of interlukin-6 (IL-6) and expression of COX-2 in bone marrow-derived mast cells [100]. This means that taxifolin could potentially be used for the treatment of allergic and inflammatory diseases [95]. Taxifolin has hepatoprotective effects because it decreases liver lesions, vacuole formation, neutrophil infiltration, necrosis and levels of malondialdehyde (MDA) which is a marker of oxidative stress and increases the activity of antioxidant enzymes [101].

Taxifolin has the ability to maintain the normal lipid profile in serum and liver of rats fed a cholesterol-rich diet. Normal lipid excretion in feces is also conserved [102]. An in vivo study also shows that taxifolin-treated animals exhibit lower levels of total liver cholesterol [103]. The neuroprotective role and anti-Alzheimer activity of taxifolin are justified by the inhibition of enzymes responsible for infection and inflammatory response in the brain stem [95].

Taxifolin acts as an antagonist on the epidermal EGFR and PI3K receptor, resulting in several effects, including antiproliferative and chemotherapeutic activity, on various cancer model systems [95,104]. It also has antiangiogenic activity proven by the in vitro inhibition of new blood vessel and branch formation [105].

#### 2.2.3. Biological Activity of *Kaempferol* and *Kaempferol* Derivatives

Kaempferol (3,5,7-trihydroxy-2-(4-hydroxyphenyl)-4H-1-benzopyran-4-one) is a tetrahydroxyflavone that has hydroxy groups located at positions 3, 5, 7 and 40. Kaempferol and its glycosylated derivatives have a variety of activities such as antioxidant, anti-inflammatory, cardioprotective, neuroprotective and anticancer [106,107]. Kaempferol has a beneficial role in different inflammatory-related illnesses such as cancers and cardiovascular and neurodegenerative diseases. Its actions result from the inhibition of inflammatory cell function and proinflammatory cytokines, chemokines and enzymes cyclooxygenase 1 (COX-1) and COX-2 [108,109]. The cyclooxygenase pathway is activated when a physical, chemical or mechanical trauma occurs at any site of the body. The arachidonic acid produced after rupture of the phospholipidic membranes is converted into prostaglandin analogues by cyclooxygenase enzymes and causes inflammation. Inhibition of the enzymes prevents this process [110]. Besides cyclooxygenase, kaempferol inhibits the expression of lipo-oxygenase (LOX) and iNOS which are also involved in inflammation [108]. This is confirmed by studies in rabbits which found that kaempferol is a potential antiatherogenic agent which prevents vascular inflammation [111]. In clinical trials, a kaempferol-rich diet significantly reduced proinflammatory cytokines such as interleukin 6 (IL-6), interleukin 8 (IL-8) and tumor necrosis factor alpha (TNF-α) which are inflammatory biomarkers [112,113]. A clinical study found that a higher intake of kaempferol is associated with a significantly decreased hazard ratio of advanced prostate cancer [114]. Kaempferol has a free radical-scavenging effect and inhibitory effect against lipid peroxidation in vitro [115]. Treatment with kaempferol heightens cell viability in response to oxidative stress, which includes unstable radicals prone to harming DNA. Kaempferol activates MAPK which can prevent the DNA damage that leads to the transformation of healthy cells into cancerous ones. Protective effects seem to only apply to normally functioning body cells while administration of kaempferol actually increases oxidative stress in cancerous glioblastoma cells by intensifying ROS production [116]. Kaempferol acts against cancer cells through several mechanisms such as inducing DNA damage and inhibiting expression of proteins associated with DNA repair in human promyelocytic leukemia HL-60 cells [117]. It also enhances cell cycle arrest in the G2/M phase in MDA-MB-231 breast cancer cells [118]. Kaempferol has chemopreventive effects against hepatocellular carcinoma by inducing autophagy, cell cycle arrest and sustained endoplasmic reticulum (ER) stress that leads to cellular damage and eventually triggers apoptosis by activating the mitochondrial intrinsic apoptotic pathway [119]. Kaempferol alone exhibits inhibitory time- and dose-dependent effects on liver cancer cell lines with little toxicity to normal hepatocytes. In the same experiment, combining kaempferol with the chemotherapeutic drug doxorubicin resulted in a stronger inhibitive effect on the viability of liver cancer cells and higher suppression of colony formation, cell cycle progression, DNA damage response and mitochondrial function [120]. The growing needs of tumors are met by the formation of new blood vessels. Vascular endothelial growth factor (VEGF) is the primary mediator of this process, termed angiogenesis [121]. Kaempferol impairs cancer angiogenesis both in vitro and in vivo through the inhibition of VEGF secretion in human cancer cell lines [122]. Kaempferol-3-O-rutinoside is an antioxidant that promotes apoptosis in cancer cells [123] and also has antifungal action against *Candida* strains [124]. Rutin, kaempferol-3-O-rutinoside and kaempferol-3-O-robinobioside have high anti-HSV-1 activity [125].

#### 2.2.4. Biological Activity of Quercetin-3-O-galactoside

Quercetin-3-O-galactoside (hyperoside) is present in plants from the genera *Hypericum* and *Crataegus* and is considered a therapeutic agent for the treatment of vascular inflammatory diseases via inhibition of the high-mobility group box 1 protein (HMGB1) signaling pathway [126]. It reduces, in a dose-dependent manner, lipopolysaccharide (LPS)-induced proliferation, migration and inflammatory responses by suppressing activation of the NF-κB signaling pathway which results in an anti-inflammatory effect in collagen-induced arthritis [127]. Hyperoside displays antifibrotic effects on cultured human hepatic LX-2 cells, which are mediated by the inhibition of NF-κB signaling and the induction of apoptosis in activated hematopoietic stem cells (HSCs). This makes hyperoside a potential candidate in the search for pharmacological agents to combat liver fibrosis [128].

#### 2.2.5. Biological Activity of Quercetin-3-O-glucoside

Querpcetin-3-O-glucoside (isoquercetrin) is one of the naturally occurring glucosides of quercetin and a predominant metabolite of quercetin in animal and human plasma [129]. Evidence shows that quercetin has great therapeutic potential in the prevention and treatment of different chronic disorders, including cardiovascular and neurodegenerative diseases, as well as cancer [130]. Isoquercetrin has potential protective effects against oxidative neuronal injuries and brain ischemia [97]. It also increases cerebral blood flow and possess antihypoxic activity [131]. Quercetin-3-O-glucoside alleviates oxidative stress, reduces ethanol-induced cytotoxicity and protects hepatic cells against ethanol-induced liver injury [132]. It is known to suppress the infiltration of pancreatic cancer cells in a dose-dependent manner. This antimigratory effect is exerted at a relatively low dose compared to other forms of quercetin [133]. Isoquercetrin exhibits significant cytotoxic action on human cervical cancer cells (HeLa) in a dose- and time-dependent manner with potent antioxidant as well as anti-inflammatory effects. Proliferation is inhibited via cell cycle arrest and apoptosis through increased generation of ROS, disruption of cellular homeostasis which eventually leads to DNA damage and cell death [134]. Quercetin-3-O-glucoside has a more potent antiproliferative effect than quercetin and quercetin-3-O-rutinoside (rutin) [135].

Quercetin and its glycosides display significant activity against the Mayaro virus [136] and the anti-influenza A virus [137]. Quercetin-3-O-glucoside is efficient against Ebola virus, both in vitro and in vivo [138], and it inhibits the replication of Zika virus in a dose-dependent manner [139]. *Streptomyces* *antibioticus* synthetizes a quercetin 3-O-glucoside derivative which is active in vitro, against numerous microorganisms such as: *S. aureus, Bacillus subtilis, Pseudomonas aeruginosa, Escherichia coli, Candida albicans, Fusarium moniliforme, Aspergillus niger* and *Aspergillus flavus* [140].

#### 2.2.6. Biological Activity of Quercetin-3-O-*rutinoside*

Quercetin-3-O-rutinoside (rutin) is a glycoside comprising the flavonol aglycone quercetin along with disaccharide rutinose and characterized by a β-glycosidic bond [141]. It possesses a broad spectrum of pharmacological activities that make it a desirable therapeutic agent in various pharmaceutical formulations or cosmeceuticals. Rutin is used for the treatment of chronic venous insufficiency [142]. It inhibits the apoptosis of cardiomyocytes and improves cardiac function in a mouse model. This effect is probably related to the restoration of the structure and function of myocardial mitochondria [143]. Rutin also reduces high blood pressure, arteriosclerosis risk and the permeability of blood vessels [144]. It is employed medicinally to reduce capillary fragility associated with some hemorrhagic diseases or hypertension in humans [145]. Due to the ability of rutin to ameliorate various neurodegenerative processes, it has been proposed as a neuroprotective compound for the treatment of Alzheimer’s disease, Parkinson’s disease and Huntington’s disease [146].

Rutin is active against UV radiation-induced damage due to its structural similarity to organic UV filters and strong antioxidant activity which can inhibit the UVB-induced inflammatory responses mediated by COX-2 and iNOS. Its ability to improve skin dermal density, reduce fine winkles and enhance elasticity make this flavonol a major ingredient in antiaging cosmetics [147,148].

The therapeutic potential of rutin is of great interest, especially as far as antiproliferative and antioxidant effects for many diseases are concerned. At micromolar concentrations, rutin possesses a dose-dependent cytotoxic effect against human melanoma cells. This is associated with a reduced viability rate, changes in cellular morphology, apoptotic-like nuclear alterations and reduced confluence [149]. Rutin can inhibit and regulate the cell cycle in the G2 and G1 phases. It stimulates apoptosis and downregulates certain oncogenic pathways including the NF-κB pathway and phosphorylation of the p38 MAPK pathway. It also inhibits growth in human glioblastoma cell lines through induction of apoptosis and cell cycle arrest in the G2/M phase, as well as regulation of expression of the pro- and antiapoptotic genes (Bcl-2, Cas-3, Bax and TP53) [150,151]. Quercetin-3-O-rutinoside increases caspase activity resulting in an apoptotic effect in prostate cancer cells [152]. It modulates various signaling pathways such as activators of transcription, MAPK, PI3K/Akt and Wnt/β-catenin signaling cascades and Janus kinase/signal transducers in carcinogenic cells. The Ras/Raf and PI3K/Akt, MAPK and TGF-β2/Smad2/3Akt/PTEN signaling pathways are stimulated through the epidermal growth factor (EGF) signaling pathway [153,154]. Rutin has been demonstrated to directly bind to the epidermal growth factor receptor (EGFR) protein and arrest the downstream signaling factors [155].

Rutin is effective in other species, not just humans. It inhibits the expression of various apoptotic markers, which may improve the health status of the mammary glands of sheep. Supplementation with rutin increases fat mobilization to provide energy and alleviate metabolic stress during the 3 weeks before calving and the 3 weeks after calving [156]. Another interesting element is the ability of rutin and its derivative rutin succinate to inhibit metalloproteinases from the venom of the Brazilian pit viper *Bothrops* *jararaca*, protecting mice from venom toxicity and ensuring their survival. These findings indicate that rutin has potential as complementary treatment for snakebites [157,158].

The antiviral activity of kaempferol, quercetin and their derivatives is well demonstrated which has resulted in several studies that investigate flavononol activity against SARS-CoV-2. These have found that administration of quercetin to COVID-19 outpatients significantly reduces the need for or the length of hospitalization, noninvasive oxygen therapy, progression to intensive care units, decreases time until virus clearance and deaths without peculiar side effects [159,160]. Rutin has the potential to induce strong inhibition against SARS-CoV-2 spike protein and main protease, resulting in a reduction in viral load [161].

### 2.3. Biological Activity of Flavan-3-ols

*S. purpurea* extracts contain two flavan-3-ols: (+)-catechin and (−)-epicatechin (Table 1). Both are antioxidants and their activity is exerted through direct mechanisms such as scavenging of ROS and chelating metal ions but also through indirect mechanisms such as induction of antioxidant enzymes, inhibition of pro-oxidant enzymes and production of phase II detoxification enzymes and antioxidant enzymes [162]. Catechins may act as therapeutic agents to inhibit oxidative damage and inflammation by suppressing oxidative stress-related pathways responsible for inflammation processes through the reduction in interleukin 5 (IL-5) and interleukin 13 (IL-13) levels [163,164,165]. Catechins inhibit oxidative damage and inflammation through the crosstalk between the MAPK and NF-κB pathways [166]. In cardiac mitochondria, epicatechin uncoupled oxidation from phosphorylation, at low concentrations stimulated phosphorylation, inhibited the respiratory chain at higher concentrations and released cytochrome c from mitochondria. These data suggest that the beneficial effects of epicatechin and its derivatives might be due to direct modulation of mitochondrial functions [167]. Catechin protects bone marrow-derived mesenchymal stem cells from oxidative stress-induced apoptosis [168]. It also influences the molecular mechanisms involved in angiogenesis, extracellular matrix degradation, regulation of cell death and multidrug resistance in cancer and related disorders [169]. As catechin showed in vitro inhibition of amyloid fibril formation [170], the two flavan-3-ols were tested in vivo either separately or in combination and reduced amyloid plaques in mouse or rat models [171]. The presence of amyloid fibrils as plaques is a key feature of several neurodegenerative diseases, in particular Alzheimer’s. This disease is characterized by amyloid aggregates formed from amyloid beta (Aβ) peptide that are deposited inside the brain or are toxic to neuronal cells [172]. Catechins and other biophenols are proposed as weapons against Alzheimer’s disease and other neurodegenerative disorders [173,174].

## 3. Monoterpenes

**Figure 3:**
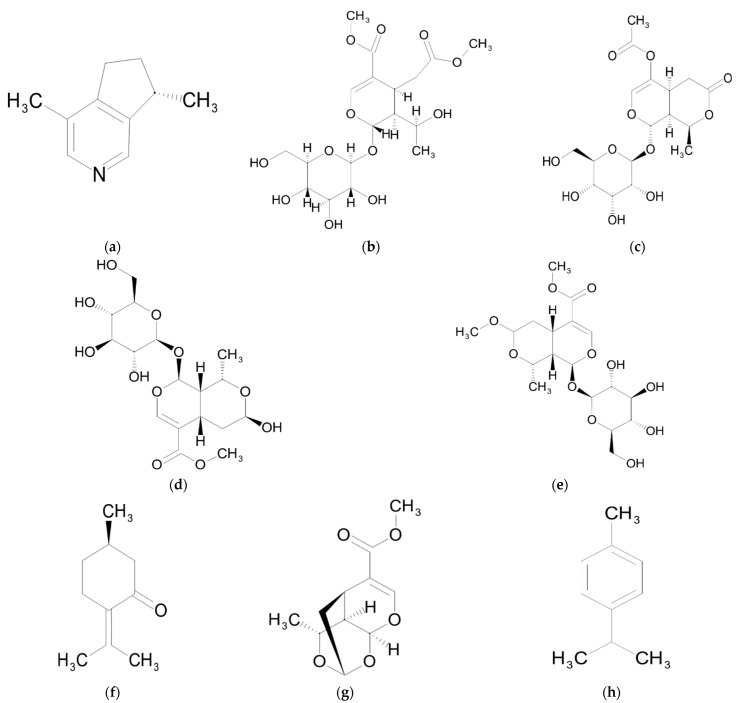
Chemical structures of monoterpenes from Sarraceniaceae species: (**a**) actinidine; (**b**) alpigenoside; (**c**) kingiside; (**d**) morroniside; (**e**) methylmorroniside; (**f**) pulegone; (**g**) sarracenin; (**h**) p-cymene.

Most of the monoterpenes identified to date in Sarraceniaceae plants belong to the class called iridoids (Table 1) which have a cyclopentane pyran structure (Figure 3) [175]. Among these compounds, sarracenin was first discovered in root extract of *S. flava* [43], in the nectaries of two *Heliamphora* species [17], in another four species, *S. alata*, *S. leucophylla*, *S. purpurea* and *S. rubra* [44], and more recently in three additional ones, *S. psittacina*, *S. purpurea* and *D. californica* [9].

### Biological Activity

Sarracenin shows significant antimicrobial activities against *S. aureus*, *Streptococcus pyogenes*, *Shigella dysenteriae*, *Klebsiella pneumonia*, *Candida albicans*, *Candida tropicalis*, *Candida thrusei* and *Candida stellatoidea* [176]. Morroniside, which was identified in *S. alata* [42], has been investigated extensively. It has therapeutic effects in multiple organs and their systems [177,178]. Morroniside shows therapeutic results in rats after acute myocardial infarction by promoting angiogenesis and improved heart function [179]. It also prevents apoptosis of cardiomyocytes induced by high glucose [180]. In the skeletal system, morroniside promotes osteoblast proliferation [181] and formation through PI3K/Akt/mTOR signaling both in vitro and in vivo [182]. Morroniside activates Akt and extracellular signal-regulated kinase (ERK) to promote cartilage cell survival and matrix synthesis [183]. It downregulates factors associated with inflammation and cartilage degradation in chondrocytes which suggests that it may be a potential protective bioactive compound against osteoarthritis [184]. 

In neurodegenerative disease, morroniside inhibits the phosphorylation of JNK, p38/MAPK and tau, and is thought to potentially aid in the treatment of Alzheimer’s disease [185,186]. Due to its antioxidant effect, morroniside improves the activity of endogenous glutathione in neuroblastoma cells. In vivo, morroniside reduces the infarct size of rats with focal cerebral ischemia and promotes the recovery of nerve functions [187]. 

Pretreatment with morroniside can reduce the production of ROS, inhibit the expression of apoptosis-related protein (Bax) and increase the expression of the antiapoptotic gene Bcl-2, to block mitochondrion-mediated apoptosis, and ultimately reduce the apoptosis of neuroblastoma cells induced by H_2_O_2_ [188]. Through its antioxidant activity, morroniside exerts neuroprotective effects in spinal cord injury and may be an effective treatment for this condition [189]. As it regulates hair follicle growth and development, partly through the Wnt/β-catenin signaling pathway, morroniside may be a potential treatment for hair loss [190].

Pulegone is a naturally occurring organic compound obtained from the essential oils of several plants from the Lamiaceae family such as *Mentha piperita* [191] that is widely used in flavoring agents, perfumery and aromatherapy [192]. In *Sarracenia*, it functions as an attractant but also as an insecticide [193] because it interferes with insect feeding behavior, development and reproduction [194]. This dual role suggests that in *Sarracenia* species it could also play a role in prey killing [13]. Pulegone shows significant antibacterial and fungicidal activity [195,196,197] as well as antihistaminic properties [198]. Pulegone exhibits anti-inflammatory activities through the regulation of NF-κB, MAPK and erythroid 2-related factor 2 (Nrf2)/heme oxygenase (HO)-1 signaling pathways. It also induces significant antioxidative effects by scavenging ROS generation in RAW 264.7 murine macrophage cells [199].

p-cymene is a volatile compound extracted from *S. alata* [45]. It shows anti-inflammatory activity, being able to modulate cytokine (TNF-α, IL-1β, IL-6) production in vitro and in vivo [200] and exhibits cytotoxic activity against a large variety of cancer cell lines such as breast, ovarian, melanoma, colorectal and hepatocellular [201].

## 4. Triterpenes

**Figure 4:**
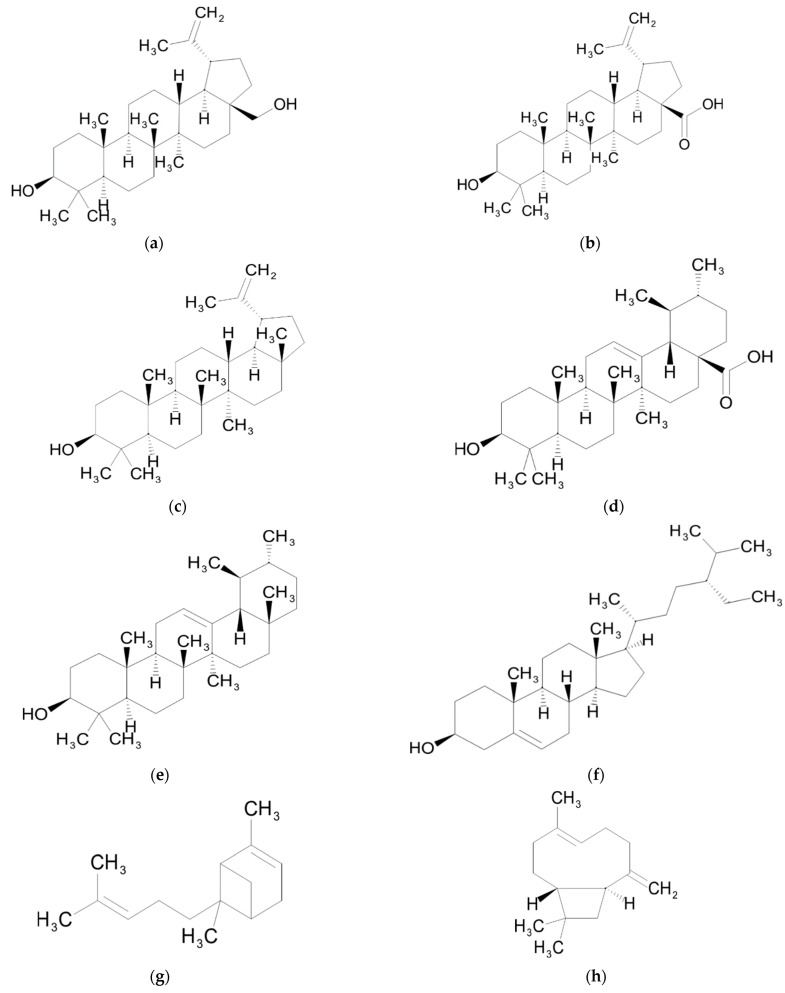
Chemical structures of triterpenes and sesquiterpenes from Sarraceniaceae species: (**a**) betulin; (**b**) betulinic acid; (**c**) lupeol; (**d**) ursolic acid; (**e**) α-amyrin; (**f**) β-sitosterol; (**g**) α-bergamotene; (**h**) β-caryophyllene.

Triterpenes are a diverse group of natural compounds widely distributed in the plant kingdom and found in leaves, stem bark, fruits and roots. They are biogenetically derived from active isoprene and the frequent object of phytochemical and pharmacological investigations [202]. The triterpenes found in Sarraceniaceae species are betulinic acid, betulin, ursolic acid, lupeol, α-amyrin and β-sitosterol (Table 1). Betulin (lup-20(29)-ene-3b,28-diol) is a lupane-type compound, characterized by an isopropylidene group and five-membered ring (Figure 4). Together with the derivative betulinic acid (3-beta-hydroxy-lup20(29)-en-28-oic acid), it is widely distributed throughout the plant kingdom [203]. In recent experiments, *S. purpurea* hairy roots induced with *Agrobacterium rhizogenes* have yielded polyphenols and triterpenes such as betulinic acid [204].

### Biological Activity

Betulin and α-amyrin have recently been found in a *S. purpurea* root extract which exhibits cytotoxicity towards the in vitro survival, migration and proliferation of mammary carcinoma cells with high tumorigenic and invasive potential [205]. Betulin and betulinic acid inhibit proliferation and induce apoptosis in various cancer cell lines such as breast [206], prostate [207], colorectal [208], leukemia [209] and lung [210]. They also manifest cytotoxic activity against multidrug-resistant tumor cells [211]. The anticancer properties of betulinic acid are associated with its ability induce apoptosis in malignant cells through both the extrinsic and intrinsic pathways [212,213]. Betulinic acid significantly represses the migration and invasion of human renal carcinoma cells in vitro and in vivo [214]. It has the ability to suppress inflammation and to regulate the immune response by modulating NF-κB activity [215] and inhibiting the cyclooxygenase pathway [216]. It can also decrease oxidative and nitrosative stress through the reduction in iNOS expression and nitric oxide [217]. Inhibition of NF-κB is also a mechanism through which betulinic acid exerts protective effects against diabetic nephropathy [218]. 

Betulinic acid protects the brains of rats from neurodegeneration and neuronal damage by diminishing the hippocampal proinflammatory cytokines and reducing oxidative stress, which results in decreased histological damage to the hippocampus [219,220]. Both betulin and betulinic acid have been tested with promising results against HIV, HSV, human papilloma virus, influenza virus and multidrug resistant bacteria [215]. The antibacterial activity of betulin, betulinic acid and ursolic acid against *E. coli*, *P. aeruginosa* and *S. aureus* is due to increased ROS generation that leads to lipid peroxidation, DNA fragmentation and bacterial death [221]. Betulinic acid has a high safety margin, as the tested dose range needed to inhibit prostate cancer growth in vitro and in vivo does not cause systemic side effects in mice [222]. Betulinic acid is toxic to neoplastic cells but only weakly toxic towards normal cells [223]. Betulin, on the other hand, shows toxicity in normal fish and murine fibroblasts [224].

Lupeol decreases the generation of proinflammatory cytokines such as TNF-α and interleukin β (ILβ) in lipopolysaccharide-treated macrophages [225]. Lupeol suppresses inflammatory mediators, and provides protection against neuroinflammation in the brains of mice [226]. The antitumor effects of lupeol are associated with its potential to modulate signaling pathways such as NF-κB and the PI3K/Akt pathway which are reported to play an important role during tumorigenesis. Lupeol kills prostate cancer cells and spares normal prostate epithelial cells, with the targeted pathways being Wnt/β-catenin signaling and Fas receptor apoptotic machinery [227,228].

Ursolic acid (3-beta-3-hydroxy-urs-12-ene-28-oic-acid) is a lipophilic pentacyclic triterpenoid [229]. As it influences cell signaling pathways, inhibits enzyme activity, induces apoptosis and reduces tumor growth, ursolic acid is considered a promising compound for cancer prevention and therapy [230]. It displays significant antitumor effects by suppressing cell proliferation and inducing cell cycle arrest in gallbladder carcinoma cells, both in vitro and in vivo [231]. Ursolic acid has antihyperglycemic action mediated through insulin secretion and an insulinomimetic effect on glucose uptake. It also stimulates synthesis, and translocation of glucose transporter protein GLUT4 by a mechanism of crosstalk between calcium and protein kinases. These effects make ursolic acid a potential antidiabetic agent with pharmacological properties for insulin resistance and diabetes therapy [232]. This triterpene shows potent activity against several bacterial species such as *S. aureus*, *Enterococcus faecalis*, *S. mutans, S. sobrinus* and *Mycobacterium tuberculosis* [233,234,235]. Unfortunately, low solubility and stability of ursolic acid in aqueous medium hinder its therapeutic application and require the development of water-soluble formulations [230,236]. 

Ursolic acid, betulin, betulinic acid, lupeol and other pentacyclic triterpenes increase glucose absorption, insulin secretion and glucose uptake in peripheral organs, contributing to the management of diabetes and diabetes-induced complications such as vascular dysfunction, retinopathy and nephropathy [237].

α-amyrin does not exhibit a strong antiproliferative activity against ovarian, pancreatic and stomach cancer cell lines [238]. It stimulates proliferation of human keratinocytes but does not protect them against UVB damage [239]. Together with its isomer β-amyrin, α-amyrin has peripheral and central analgesic effects independent of the opioid system, and also shows a potent anti-inflammatory activity [240].

β-sitosterol, a bioactive phytosterol, is naturally present in plant cell membranes. Its chemical structure is similar to the mammalian cell-derived cholesterol [241]. β-sitosterol reduces oxygen free radicals and hydrogen peroxide levels in RAW 264.7 cells and stimulates enzymatic antioxidants. It also reverts the impairment in glutathione/oxidized glutathione ratio, leading to the conclusion that phytosterol can scavenge ROS [242]. Due to its potency as an antioxidant and hypolipidemic agent, treatment with β-sitosterol decreased the levels of stress and lipid parameters, resulting in normal levels of insulin receptor (IR) and GLUT4 in diabetic rats fed with a high-fat diet [243]. β-sitosterol has anti-inflammatory properties, being able to inhibit phosphorylation of NF-κB and the activity of this transcription factor in macrophage cells [244].

## 5. Sesquiterpenes

Among the sesquiterpenes found in *S. alata* (Table 1), the predominant ones are β-caryophyllene and α-bergamotene (Figure 4) [45]. β-caryophyllene is a volatile compound of essential oils from many herbs and spices [245] including *Cannabis sativa* [246]. 

### Biological Activity

The ability of β-caryophyllene to bind to cannabinoid receptor type 2 (CB2) stimulates both MAPK and PI3K signaling pathways which results in anticancer and analgesic properties [247]. Due to this selectivity towards CB2, it has applications for various pathological conditions such as nervous system disorders and various inflammatory diseases (rheumatoid arthritis, atherosclerosis) [248].

α-bergamotene is one of the main components of essential oil extracted from *Citrus medica* fruit which has high antioxidant and antimicrobial activity against Gram-positive bacteria (*Bacillus subtilis, Micrococcus luteus, Staphylococcus aureus*), Gram-negative bacteria (*Escherichia coli*) and yeast (*Saccharomyces cerevisiae*) [249]. 

## 6. Fatty Acids

**Figure 5:**
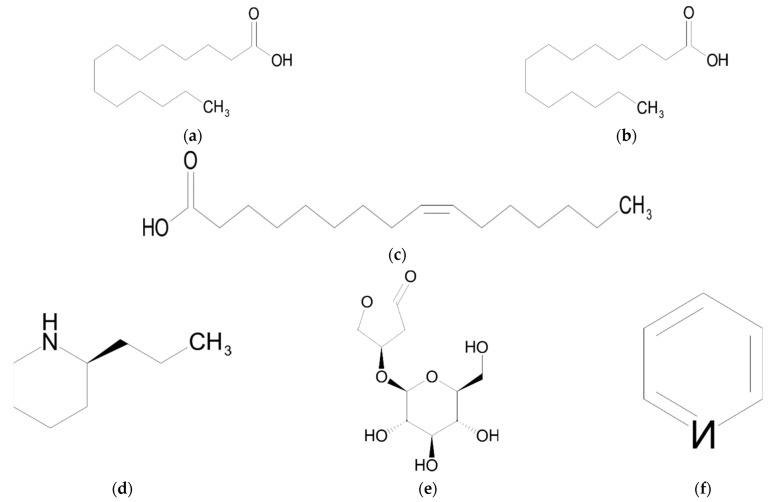
Chemical structures of fatty acids and other compounds from Sarraceniaceae species: (**a**) tetradecanoic acid; (**b**) hexadecanoic acid; (**c**) (Z)-9-hexadecenoic acid; (**d**) coniine; (**e**) goodyeroside; (**f**) pyridine.

The carboxylic or fatty acids found in Sarraceniaceae species are tetradecanoic (myristic acid), hexadecanoic (palmitic acid) and (Z)-9-hexadecenoic (palmitoleic acid) (Figure 5) which function as attractants for insects [9].

### Biological Activity

Palmitic acid has been proposed as an agent with anticancer activities because of its ability to inhibit topoisomerase I and induce autophagy in leukemic cells and lung cancer cells. Topoisomerases play a central role in DNA metabolism by cutting and rejoining one or both strands of the duplex DNA. Inhibitors of these enzymes have the ability to kill cells undergoing DNA replication or transcription or experiencing repair of DNA damage. Since cancer cells divide much more rapidly than normal cells, they are the most likely victims of topoisomerase inhibitors [250,251].

Cytotoxic effects of palmitic acid alone or in combination with other fatty acids have also been observed in breast cancer cells [252], hepatocyte carcinoma cells [253] and colon carcinoma cells [254,255].

On the other hand, fatty acids do not always have a beneficial effect on health. Both palmitic acid and myristic acid can compromise cell response and repair pathways induced by DNS damage in primary cells (fibroblasts and osteoblasts) but not in colon or breast cancer cells [256]. Furthermore, a palmitic acid-rich diet promotes colorectal cancer cell growth in vitro and in vivo [257] and induces stable transcriptional and chromatin changes that lead to a long-term stimulation of metastasis in mouse oral carcinomas and melanoma [258]. Palmitic and myristic acid can act together to induce lipotoxicity and nonalcoholic steatohepatitis in mice [259]. In exogenous applications, fatty acids are common raw materials in cosmetics [260]. Long chain fatty acids, in particular, can reduce the loss of moisture on the skin surface, resist the entry of harmful substances and maintain normal skin barrier function [261]. 

In mice fed a high-fat diet, the unsaturated palmitoleic acid reduces hepatic gluconeogenesis by decreasing the expression of sirtuin 3 (SIRT3), a protein deacetylase and the activities of gluconeogenic enzymes, such as phosphoenolpyruvate carboxykinase (PEPCKP), pyruvate carboxylase (PC) and malate dehydrogenase 2 (MDH2) [262]. It also acts as an anti-inflammatory mediator [263,264] and displays antibacterial properties against *Staphylococcus aureus* [265].

## 7. Alkaloids

Coniine is a piperidine alkaloid originally found in *Conium* *maculatum* (hemlock) [9]. It is a neurotoxin that causes death from respiratory failure [266]. Among carnivorous plants, it was first isolated from *S. flava* (Table 1) and is considered to be an insect paralyzing agent [48]. Coniine has significant effects on presynaptic nicotinic acetylcholine receptors. These could make it useful as a presynaptic nicotinic receptor antagonist in pharmacological studies [267]. It could be employed particularly for pain relief without an addictive side effect [268].

## 8. Other Compounds

Goodyeroside is the epimer of the better known compound kinsenoside (Figure 5). First isolated from *Goodyera* species [269], it has been found in *S. purpurea* [26,41]. The current interest in this substance is due to its hepatoprotective effect on liver injury induced by CCl4 in primary cultured rat hepatocytes [269]. Goodyeroside also suppresses inflammation through inhibition of the NF-κB signal pathway [270]. 

Pyridine is a volatile compound from *S. alata* [45]. It is a scaffold that has been consistently incorporated in a diverse range of drug candidates to sharpen their potency and to improve pharmacokinetics [271].

## 9. Conclusions and Future Directions

Although rather poorly investigated, Sarraceniaceae plants contain a wealth of potentially useful secondary metabolites. These have anti-inflammatory, anticancer, antimicrobial, antiviral, cardioprotective, hepatoprotective or neuroprotective abilities proven both in vitro and in vivo (Figure 2). This review is significant because it synthetizes current knowledge about secondary metabolites from Sarraceniaceae species and explores potential uses stemming from their biological activity. The review also highlights how little information is available about the chemical composition and the in vitro propagation of plants from the *Heliamphora* genus. 

For future directions, this overview can be used as the starting point for further research that should develop in two main directions: identification of new useful metabolites and increasing the production of known ones. Identification of novel compounds and alternative plants uses can be carried out in cost-effective ways by using phylogenetic approaches [272,273] which have not been employed to date in Sarraceniaceae. Increasing the production of natural compounds raises the problem of producing substantial quantities of biomass for extraction. Plants belonging to the Sarraceniaceae family have a limited natural habitat (*Darlingtonia californica,* *Heliamphora*) or are endangered (*S. alabamensis, S. jonesii, S. oreophila*) in the wild because of habitat destruction and poaching or over-collecting. That is why the quantities of plant material required by the pharmaceutical industry for the manufacturing of products with commercial potential should be sourced from either in vivo propagation methods or micropropagation. Both types of techniques result in large amounts of biomass at relatively low cost. Both have the advantage of introducing plant tissue to genetic transformation and elicitation that can be employed separately or together and have been extensively developed for other species. In this context, another direction for future research is the development and improvement of micropropagation protocols for Sarraceniaceae species to supplement the small number of currently published ones [45,274,275,276]. Genetic transformation has already been carried out in *Dionaea muscipula* [277], *Drosera capensis* [278,279], *Drosera rotundifolia* [280,281] and *Nepenthes mirabilis* [282] and its application looks promising for producing betulinic acid in *Sarracenia purpurea* [204]. The production of recombinant proteins in *Sarracenia purpurea* has already been suggested [283]. Elicitation resulted in high levels of antimicrobial secondary metabolites for *Dionaea muscipula* [277] and *Nepenthes khasiana* [284]. Applying these techniques would decrease the cost of the secondary metabolites themselves, making them more accessible to the market. 

In summary, this review provides further support that plant secondary metabolites from Sarraceniaceae species are useful in the fight against various diseases and spotlights the need for future investigations into their biochemistry and the production of pharmaceutically and commercially valuable compounds from their tissues.

## Figures and Tables

**Table 1:** Secondary metabolites detected in plants from the Sarraceniaceae family.

Chemical Class	Compound	Reference
Flavonoids	Cyanidin	[34]
	Delphinidin	[34]
	Taxifolin (dihydroquercetin)	[39]
	Taxifolin-3-O-glucoside	[26]
	Taxifolin-7-O-galactoside	[39,40]
	Gossypetin-3-O-galactoside	[39]
	Isorhamnetin-3-O-glucoside	[26]
	Kaempferol-3-O-(6″-caffeoylglucoside)	[26]
	Kaempferol-3-O-rutinoside	[26,40]
	Quercetin 3-O-α-L-arabinopyranoside	[35]
	Quercetin-3-O-arabinoside	[39]
	Quercetin-3-O-galactoside (hyperoside)	[26,39,40,41]
	Quercetin-3-O-glucoside (isoquercetrin)	[40]
	Quercetin-3-O-rutinoside (rutin)	[26]
	Tamarixetin-3-O-galactoside	[26,39,40]
	(+)-Catechin	[40]
	(−)-Epicatechin	[40,41]
Monoterpenes	7α-O-methylmorroniside	[26,42]
	7β-O-methylmorroniside	[26,42]
	Actinidine	[9]
	Alatenoside	[42]
	Alpigenoside	[9,42]
	Kingiside	[42]
	Morroniside	[26,41,42]
	Pulegone	[9]
	Sarracenin	[9,43,44]
	p-cymene	[45]
Triterpenes	Betulin	[46]
	Betulinic acid	[39,40,46]
	Lupeol	[47]
	Ursolic acid	[39,40]
	α-amyrin	[47]
	β-sitosterol	[47]
Sequiterpenes	α-bergamotene	[45]
	β-caryophyllene	[45]
Carboxylic acids (fatty acids)	Tetradecanoic acid (myristic acid)	[9]
	Hexadecanoic acid (palmitic acid)	[9]
	(Z)-9-hexadecenoic acid (palmitoleic acid)	[9]
Alkaloids	Coniine	[9,48]
	Lagumicine	[9]
Other compounds	(Z)-13-docosenamide (erucamide)	[9]
	6′-O-caffeoylgoodyeroside	[26]
	Goodyeroside	[26,41]
	Nonanal	[9]
	Pyridine	[45]
